# Euryhaline Atlantic stingray (*Hypanus sabinus*) exhibit elevated oxygen supply capacity in hyposaline water: implications for estuarine species resilience and conservation

**DOI:** 10.1093/conphys/coaf071

**Published:** 2025-10-21

**Authors:** Sophia M Emmons, Jodie L Rummer, Joshua P Kilborn, Maria A Pierce, Alexander W Timpe, Colin A Simpfendorfer, Brad A Seibel

**Affiliations:** College of Science and Engineering, James Cook University, 1 James Cook Dr., Douglas , QLD 4811, Australia; College of Marine Science, University of South Florida, 830 1st St S, St. Petersburg, FL 33701, USA; College of Science and Engineering, James Cook University, 1 James Cook Dr., Douglas , QLD 4811, Australia; College of Marine Science, University of South Florida, 830 1st St S, St. Petersburg, FL 33701, USA; College of Science and Engineering, University of North Carolina Wilmington, Friday Hall, Suite 2001, 601 S. College Road, Wilmington, NC 28403-5642, USA; College of Marine Science, University of South Florida, 830 1st St S, St. Petersburg, FL 33701, USA; College of Science and Engineering, James Cook University, 1 James Cook Dr., Douglas , QLD 4811, Australia; College of Marine Science, University of South Florida, 830 1st St S, St. Petersburg, FL 33701, USA

**Keywords:** Coastal habitat, elasmobranch, estuary, hypoxia, metabolic rate, respirometry, salinity

## Abstract

Estuarine environments are characterized by fluctuating abiotic conditions, such as salinity and oxygen partial pressure, which challenge the physiological systems of resident species. Organisms inhabiting these systems have evolved physiological plasticity to cope with this variability, particularly in relation to oxygen availability. Estuarine species tend to exhibit greater hypoxia tolerance compared to coastal marine species, likely due to periodic low oxygen exposure. However, the effects of salinity fluctuations on oxygen transport remains unclear. This study investigated the effects of different salinity levels on the oxygen supply capacity of the Atlantic stingray (*Hypanus sabinus*), a euryhaline elasmobranch in the temperate west Atlantic and Gulf of Mexico. Maximum metabolic rates and oxygen supply capacity were measured at high, medium and low salinities (32, 16 and 6 psu, respectively). Critical oxygen pressure (*P*_cMax_), where maximum metabolism and aerobic scope become oxygen limited, was also calculated. Results showed a significant 20% increase in oxygen supply capacity and a 30% decrease in *P*_cMax_ under low salinity compared to high salinity. These findings suggest that Atlantic stingrays improve their oxygen supply capacity and are more hypoxia tolerant in hyposaline conditions. Enhanced oxygen supply capacity may represent an adaptive strategy, enabling Atlantic stingrays to maintain metabolic performance in low oxygen environments. This study provides novel insight into the adaptive capacity of euryhaline elasmobranchs to balance oxygen transport and metabolic function across salinity gradients. It highlights the importance of physiological plasticity in estuarine species’ responses to climate-driven changes in salinity and oxygen availability. These findings can inform management strategies by identifying species with greater resilience to hypoxia and salinity shifts, supporting more effective conservation efforts under future climate scenarios.

## Introduction

Estuaries are dynamic ecosystems where abiotic factors such as salinity, temperature and dissolved oxygen fluctuate naturally on diel, tidal and seasonal cycles. Temporal variation in precipitation and temperature creates complex habitats that are crucial for many species and across life stages (Able, 2005; Barbier *et al.*, 2011; Norton *et al.*, 2012). Low salinity environments, typical of estuaries, provide essential resources such as nursery grounds, abundant prey and protection from predators for euryhaline and estuarine species (Heupel *et al.*, 2007; Heupel and Simpfendorfer, 2011; Grant *et al.*, 2019). Estuarine organisms are likely to encounter increasing salinity fluctuations as coastal development and climate change intensify environmental variability (Kennish, 2023; Valle-Levinson and Li, 2023). Rising temperatures are expected to exacerbate eutrophication, leading to more frequent and severe hypoxic events in estuaries (Selman *et al.*, 2008; Altieri and Gedan, 2015). Additionally, coastal regions experience fluctuating salinities due to saltwater intrusion, drought and changes in freshwater inflows driven by regional precipitation variability (van der Wiel and Bintanja, 2021; Costa *et al.*, 2023; Valle-Levinson and Li, 2023). In the Gulf of Mexico, estuaries frequently undergo seasonal anoxia from eutrophication, resulting in periods of low coastal salinity, high temperatures and oxygen depletion (Rabalais *et al.*, 2001, 2002; Breitburg *et al.*, 2018). These compounded stressors may have cascading effects on the physiological performance of estuarine species, increasing oxygen demands while simultaneously altering metabolic processes in response to environmental shifts.

Species inhabiting these environments have evolved physiological adaptations that allow them to cope with natural, cyclical variability in their environment (Hazon *et al.*, 2003; Madeira *et al.*, 2012; Marshall, 2012; Seibel, 2023). However, regional and global anthropogenic impacts, such as changes in land use and altered precipitation patterns, disrupt or increase the intensity of fluctuations in their abiotic environment (Gillanders and Kingsford, 2002; van der Wiel and Bintanja, 2021). Alterations to freshwater input and eutrophication from human impacts can affect salinity and dissolved oxygen levels, potentially pushing these variables beyond the physiological tolerance limits of estuarine organisms (Smith, 2003; van der Wiel and Bintanja, 2021; Constance *et al.*, 2024). When such changes occur, species may be unable to utilize essential resources in these critical habitats, thus threatening their survival.

**Figure 1:**
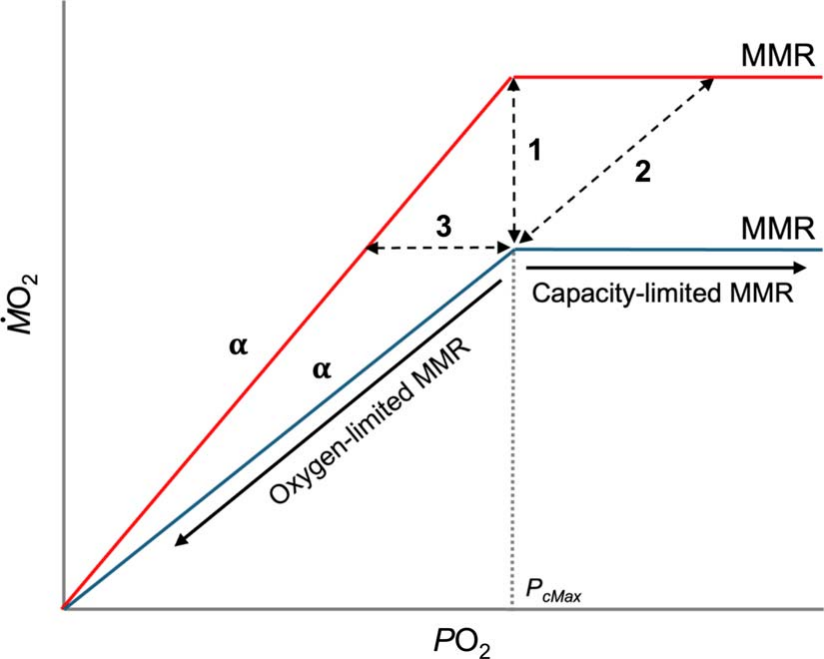
Schematic representation of the oxygen (*P*O_2_) dependence of MMR under two hypothetical salinity treatments (red and blue). The *P*_cMax_ (dotted line) is the *P*O_2_ below which MMR becomes limited by ambient oxygen and above which it is limited by evolved physiological capacity. Oxygen supply capacity (α) is calculated as MMR/*P*O_2_ below *P*_cMax_ and is the slope of the oxygen-limitation lines. An increase in MMR (**1**) may be met by improved oxygen supply capacity or an increase in *P*_cMax_ (**2**). An increase in oxygen supply capacity with no increase in MMR (**3**) will result in a decrease in *P*_cMax_, indicating improved low oxygen tolerance.

We can assess how organisms respond to different environmental variables, such as temperature, salinity and oxygen availability by measuring changes in their oxygen uptake rate, which is a proxy for total metabolic rate (*Ṁ*O_2_), under different conditions (Fry, 1971; Chabot *et al.*, 2016; Alfonso *et al.*, 2021). Below a critical oxygen partial pressure (*P*_cMax_), maximum metabolic rate (MMR) is oxygen limited (Fig. 1; see Box 1). The *P*_cMax_ varies across species, but in environments with relatively stable oxygen levels, such as the coastal subtidal ocean, it is typically near the prevailing *P*O_2_ (Seibel and Deutsch, 2020). For example, coastal marine species experience oxygen-limited metabolism near 21 kPa, which corresponds to the fully air-saturated seawater conditions they generally inhabit (Seibel and Deutsch, 2020). In contrast, oxygen levels in estuarine environments are more variable. Species tend to become oxygen-limited near the estuary’s mean daily *P*O_2_ level, which is often about half air-saturated (mean *P*_cMax_ = 11.5 kPa; Seibel, 2023). This indicates that estuarine species are more hypoxia tolerant when compared to their coastal counterparts, as they can sustain MMR at significantly lower oxygen levels, about half air-saturation, where the metabolic capacity of most coastal species would be severely limited.

**Box 1.** Identifying symbols and definitions of common terms used in this study.

Terms	Symbols	Definition
Oxygen supply capacity	α	α is the maximum amount of oxygen that can be supplied per unit time and oxygen pressure (μmol O_2_ g^−1^h^−1^kPa^−1^; Seibel *et al.*, 2021). It is calculated as the maximum achievable metabolic rate at a given *P*O_2_ (α = MMR/*P*O_2_).
Instantaneous oxygen supply capacity	α_0_	The oxygen supplied per unit *P*O_2_ at any given instant (α_0_ = MR/*P*O_2_) increases as oxygen declines or metabolic rate (MR) increases until it reaches its maximum capacity at *P*_crit_ (α = MR/*P*_crit_).
Metabolic rate Maximum metabolic rate Standard metabolic rate	MR MMR SMR	The rate of aerobic energy usage, estimated from oxygen consumption. MMR is the highest achievable MR, typically measured during or following exercise protocols. At *P*O_2_ less than *P*_cMax_, MMR is directly oxygen dependent (MMR = *P*O_2_ • α). SMR is the fasted, resting MR at a specified temperature.
Oxygen partial pressure	*P*O_2_	The portion of the pressure exerted by gas that is attributable to oxygen, expressed relative to what seawater would hold in equilibrium with air. At saturation, oxygen is always 21% of the total, or ~21 kPa (101 kPa = 1 atm). Oxygen concentration [O_2_] depends on *P*O_2_ and gas solubility, which is dependent on temperature and salinity.
Hypoxia		In the context of this study, hypoxia is defined as the point at which *P*O_2_ is below *P*_cMax_ and maximum achievable MR is limited by available oxygen. For many shallow water marine species, this occurs when *P*O_2_ is below 21 kPa.
Over saturation		In the context of this study, over-saturation is defined as the *P*O_2_ beyond *P*_cMax_, when metabolic rate is capacity limited. For many shallow water marine species, this occurs when *P*O_2_ is above air saturation (21 kPa).
Aerobic scope Absolute aerobic scope Factorial aerobic scope	AS AAS FAS	The absolute (AAS) or factorial (FAS) difference between standard and MMR that represents the oxygen available to support all aerobic energetic activities, including locomotion, growth and reproduction.
Critical *P*O_2_ *P*_crit_ for SMR *P*_crit_ for MMR	*P*_crit_ *P*_cSMR_ *P*_cMax_	The *P*O_2_ at which oxygen supply reaches its maximum capacity (α) for any given MR (*P*_crit_ = MR/α). *P*_crit_ is any rate-specific point on the oxygen-limiting line defined by α. Equivalently, *P*_crit_ is the *P*O_2_ below which its corresponding MR becomes oxygen limited. Throughout this paper, *P*_crit_ specified for a particular MR is indicated by a subscript (e.g. *P*_cSMR_ or *P*_cMax_, for SMR and MMR, respectively).

Estuarine species’ ability to sustain MMR at lower oxygen levels stems from an enhanced capacity to supply oxygen (Seibel and Deutsch, 2020). Oxygen supply capacity (α) is a species- and temperature-specific constant that describes how effectively oxygen is delivered to the tissues, encompassing all mechanisms that impact oxygen uptake and delivery, such as ventilation, gill-surface area, cardiac output and blood-oxygen binding (Seibel *et al.*, 2021; Wagner, 2023). The α may change, via alterations in any or all steps in the oxygen cascade, with acclimation conditions to meet changing energy demands or environmental conditions. When considered together, α and MMR inform *P*_cMax_ (Eq. (1)), the *P*O_2_ below which an organism’s aerobic scope becomes limited by available oxygen. Thus, *P*_cMax_ is impacted by changes in MMR and α, which are both modulated by environmental conditions. For example, oxygen supply typically increases with rising temperatures to support elevated demand at the prevailing *P*O_2_ (Fig. 1; Alfonso *et al.*, 2021). If α does not increase to keep pace with changing demands, metabolism becomes oxygen-limited under those conditions (Seibel and Deutsch, 2020). Environmental tolerance limits are often attributed to a failure of oxygen supply to match this demand, but this is contentious (Pörtner *et al.*, 2017; Jutfelt *et al.*, 2018; Seibel and Deutsch, 2020). While salinity has been shown, in some instances, to influence individual steps in the oxygen supply chain, such as blood-oxygen binding and cardiac output (Nikinmaa and Salama, 1998; Ern *et al.*, 2014; Sundell *et al.*, 2018), its effect on total oxygen supply (i.e. α) has not been quantified. The costs of osmoregulation are estimated to account for 0–30% of the standard metabolic rate (SMR) in teleost fishes, with euryhaline species on the lower end, and 10–15% in elasmobranchs (Kirschner, 1993; Ern *et al.*, 2014; Ern and Esbaugh, 2018). The impact of salinity on oxygen supply capacity is a critical gap in understanding how estuarine species adjust physiologically to fluctuating environmental conditions.

**Table 1:** Collection and assessment data for Atlantic stingrays (*H. sabinus*) tested at each salinity treatment (*N* = 18)

Salinity (psu)	32	16	6
*n*	6	6	6
Mass (g)	477.67 ± 392.18	407.33 ± 315.28	807.11 ± 121.18
Sex (F:M)	4:2	3:3	0:6
Collection period (2023)	July–September	October–November	November–December
Chamber volume (L)	40 (×2), 25 (×2), 9 (×2)	40 (×2), 9 (×4)	40 (×6)

Numbers in parentheses indicate the number of individuals that were tested in each respirometry chamber size. Mean ± standard deviation.

The Atlantic stingray (*Hypanus sabinus*), a euryhaline elasmobranch found throughout temperate and subtropical waters of North America and the Gulf of Mexico (Last *et al.*, 2016; Grant *et al.*, 2019; Constance *et al.*, 2024), presents an ideal model species for investigations into the impact of salinity changes on metabolic rate and α, which interact to establish critical oxygen limits. This species thrives in environments ranging from fully marine to freshwater, including estuaries (Gunter, 1938; Snelson *et al.*, 1988; Johnson *et al.*, 1996; Piermarini and Evans, 1998). Its ability to inhabit environments with such variable salinity makes the Atlantic stingray an ideal candidate to investigate how euryhaline elasmobranchs cope with environmental shifts. In this study, we aimed to estimate MMR and oxygen supply capacity across three salinities representative of the species’ natural range to assess how these physiological parameters are affected by salinity changes. The findings of this research will provide valuable insight into how environmental fluctuations, particularly in salinity and oxygen, may challenge species residing in increasingly altered estuarine habitats.

## Materials and Methods

### Specimen collection and captivity

Atlantic stingrays (*N* = 18, 7F:11M; Table 1) were collected from coastal waters off Pinellas County, Florida at Weedon Island (27.847º, −82.616º) and Lassing Park (27.751º, −82.627º) from July to December 2023. Individuals were captured close to shore using either a dipnet or a 6 × 2 m seine net (2 mm mesh), and collections occurred under a Special Activities Licence granted by the state of Florida (SAL-22-2143-SR). Once captured, individuals were transported to holding tanks (~550 L) connected to a recirculating system with a maximum capacity of ~ 9,430 L supplied with artificial seawater at the University of South Florida, College of Marine Science aquarium facilities. System temperature was maintained around 25°C (mean 24.50°C ± 0.99 SD) via whole-system inline heater and ambient room temperature control and salinity was maintained at 32 psu. Water quality of each holding tank as well as the entire system was tested daily for nitrate, nitrite, ammonia and pH levels (API Saltwater Master Test Kit, Chalfont, USA); water exchanges of approximately 500 L occurred weekly for the full system or when the aforementioned tests exceeded healthy conditions (nitrate ≤ 80 ppm, nitrite ≤0.5 ppm, ammonia ≤0.5 ppm, 7.7 ≤ pH ≤ 8.2). Individuals were acclimated to captive conditions before undergoing experimental trials, which was the point when an individual ate consistently (1–1.5 weeks). Stingrays were fed every second day fresh and pre-frozen shrimp to satiation. All experiments were outlined and approved by the Institutional Animal Care and Use Committee (IACUC) under protocol #W IS00011888.

### Experimental design

**Figure 2:**
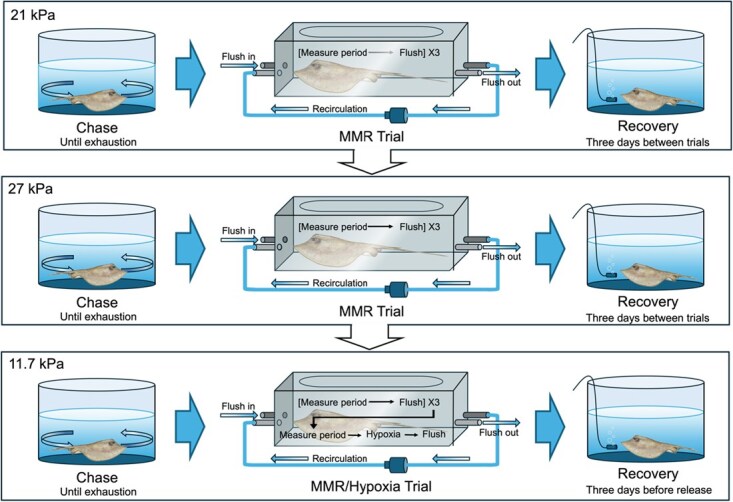
Flow chart of respirometry trial procedure for each stingray at its experimental salinity. Prior to each trial, the test stingray was chased until exhaustion, given one minute of air exposure, and placed into the sealed chamber. Individuals were tested across three target *P*O_2_ points (21, 27 and 11.7 kPa) to determine MMR, as observed across three measurement periods. Following the third measurement period of the 11.7 kPa trial, the chamber remained sealed and transitioned into a hypoxia trial, continuing until the stingray reached critical oxygen limit (*P*_crit_). The oxygen uptake data from the hypoxia portion of the trial were used to establish oxygen supply capacity. After each trial, the test stingray recovered for three days, which included re-acclimation to 32 psu and a feeding event. Stingray illustration by Sophia Emmons*.*

This study used intermittent-flow respirometry to determine how different salinities impact MMR and α. Due to small sample size and facility limitations, three target salinities were chosen to represent the Atlantic stingray’s natural range: high salinity (32 psu; *n* = 6), medium salinity (16 psu; *n* = 6) and low salinity (6 psu; *n* = 6). Temperature was held constant at 26.0° (± 0.22 SD) to eliminate its potential effects on metabolic rate. Each stingray was tested at a single salinity and not reused across salinities. At its target salinity, it underwent three MMR respirometry trials, one at each *P*O_2_ (in subsequent order: 21.0, 27.0 and 11.7 kPa), the latter of which continued into a hypoxia trial form 11.7 kPa (Fig. 2). Each stingray was tested at a single salinity and not reused across salinities (acclimation process described below). Starting *P*O_2_ values were chosen to establish *P*_cMax_, the *P*O_2_ below which maximum metabolism becomes oxygen dependent (Fig. 1). The starting oxygen levels employed are within the range of natural oxygen fluctuations in estuaries. Between trials and regardless of salinity treatment, stingrays were fed and re-acclimated to high salinity over the course of 12 h and maintained for 48 h before undergoing subsequent salinity reconditioning prior to the next respirometry trial. After their final trial, stingrays were reacclimated to 32 psu and ultimately released at the site of their capture.

### Experimental set up

Before each experiment, the temperature bath (~500 L), which held the respirometry chambers, was filled with water at the experimental salinity and temperature (26°C). Water was filtered with a 1-μM filter and an in-line ultra-violet sterilizer. Salinity was adjusted to the target using a mix of main-system seawater (32 psu) and deionized water, and the experimental temperature was achieved using aquarium heaters. For each trial, one of three acrylic respirometry chambers (9, 25 or 40 L), plumbed for intermittent water flow and selected based on the stingray’s size (Svendsen *et al.*, 2016), was submerged in the temperature bath. Each chamber was equipped with a circulating pump to mix the sealed chamber’s contents and a flush pump (Syncra Silent Multifunction Pump, Sicce S.r.l, Prozzoleone, Italy) to replenish the chamber with water from the temperature bath (Svendsen *et al.*, 2016). Before each trial, the chamber water was flushed to reach the target oxygen saturation level (21, 27 and 11.7 kPa). Target oxygen saturation was achieved via gas bubbling using air stones and an anoxic (99% N_2_) or hyperoxic (50% O_2_) gas mix. The oxygen content (% air saturation) was measured continuously throughout the trial using a Firesting Optical Oxygen and Temperature Meter (PyroScience gmbh, Aachen, Germany).

### M‌MR and oxygen supply capacity

Prior to each trial, individuals were fasted for 48 h to ensure a post-absorbative state (Di Santo and Bennett, 2011). A lack of faecal matter observed in the respirometry chambers after each trial further evidenced the post-absorbative state. For each salinity treatment, stingrays were isolated from the main system while salinity was gradually reduced to the target salinity over 12 h. Stingrays were then maintained at their target salinity for 24 h prior to each experimental trial. Immediately preceding each trial, the test individual underwent a combination of exhaustive procedures meant to elicit its maximum rate of oxygen uptake (MMR; Reidy *et al.*, 1995). In a circular holding tank at the target salinity, the individual was chased with a 3-m PVC pole by gently prodding the pelvic fins to elicit quick, exhaustive movements until it no longer responded to touch. The stingray was then inverted dorsal-side down and allowed to right itself. This process was repeated until stingray could no longer right itself. Then, it was given 1 min of air exposure before being placed inside the chamber (Roche *et al.*, 2013). The first measurement period of the MMR trial was initiated immediately after the stingray was sealed in the respirometry chamber following the exhaustive procedure and air exposure. MMR trials consisted of three 15-min measure periods, during which time the chamber remained closed. Between measurement periods, the chamber’s flush valves were opened, allowing clean water from the temperature bath to flush through the chamber until the oxygen returned to the initial trial value (e.g. 27.0 kPa). This process took approximately 3–5 min, depending on chamber size (Table 1). Following the third measurement period, the chamber was flushed with air-saturated water. The stingray was removed, weighed and returned to its holding tank. See Fig. 2 for overview of chase, trial and recovery procedure.

Oxygen supply capacity was measured during hypoxia trials, initiated at low *P*O_2_ to increase the likelihood of reaching limiting oxygen pressures during the experiment. The initial procedure followed the same methodology as the MMR trials (section **MMR and oxygen supply capacity**) with two key differences: (i) the initial *P*O_2_ was 11.7 kPa, and (ii) the chamber was not flushed following the third measurement period. Instead, the MMR portion of the trial transitioned into a hypoxia trial. Starting at a lower *P*O_2_ ensured that the heightened metabolic rate at the beginning of the trial increased the likelihood of reaching oxygen supply capacity during the hypoxia trial period (Seibel *et al.*, 2021). The stingray remained in the sealed chamber until a critical oxygen threshold was reached, indicated by burst swimming and irregular spiracle pumping, which typically occurred once oxygen dropped below 2 kPa. The chamber was then flushed with air-saturated water, marking the end of the trial. Note that stingray respiration during closed respirometry would result in elevated *P*CO_2_ and concomitant acidification. However, the closed portion of the MMR trials was short (15 min), punctuated by flushing, minimizing any *P*CO_2_ artefacts. Following each trial, microbial oxygen uptake was measured in the empty, sealed chamber for 1 h (Rodgers *et al.*, 2016). Microbial *Ṁ*O_2_ was calculated from oxygen uptake rate during the microbial measurement period using the “respirometry” R package with a negligible value for mass (*W* = 0.0001) and then subtracted from the total *Ṁ*O_2_ measured during each trial (Birk, 2024).

### Calculating MMR, oxygen supply capacity and *P*_cMax_

For each trial, the stingray’s MMR was determined from the measurement period in which *P*O_2_ declined most rapidly (Supplementary Fig. S1). Mass-specific metabolic rate (*Ṁ*O_2_; μmol O_2_ g^−1^ h^−1^) was calculated as the slope of a linear least-squares regression using the R package “respirometry” (Birk, 2024), using consecutive bins at 45-s intervals. Metabolic rate was mass corrected to account for discrepancies in body size across the animals using the following equation:


(1) \begin{equation*} \dot{M}{\mathrm{O}}_2={cW}^b \end{equation*}

where *Ṁ*O_2_ is the mass-specific MMR (μmol O_2_ g^−1^ h^−1^) calculated for each saturation trial, *c* is a species-specific normalization constant determined using nonlinear least squares regression, *W* is the body mass (g) of the animal and *b* is the mass scaling coefficient. The coefficients were calculated in the “respirometry” R package (Birk, 2024) using the average mass (g) from the 21 kPa saturation group as the common mass after mass re-scaling (mean 594.1 g ± 367.3 SD) because this measurement was taken after the first trial each stingray underwent.

**Figure 3:**
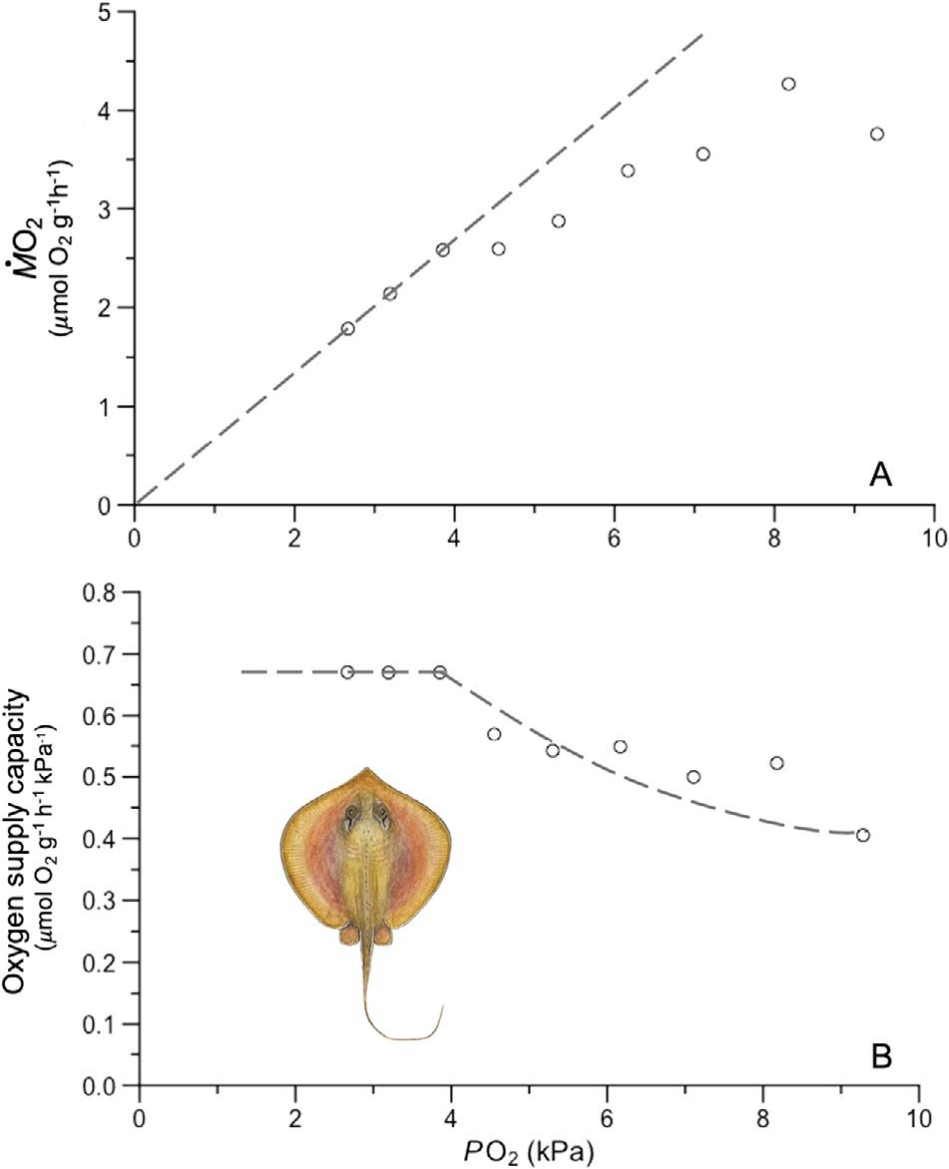
Representative hypoxia trial from one individual at low salinity (6 psu). (**A)** Oxygen uptake rate (*Ṁ*O_2_) of the stingray declines with oxygen partial pressure (*P*O_2_) as available oxygen is removed from the closed respirometry chamber by the stingray. (**B)** As *P*O_2_ declines, the ratio of *Ṁ*O_2_ to *P*O_2_ increases until a consistent value is reached, indicating that the stingray has reached its oxygen supply capacity (α). The dashed line indicates oxygen supply capacity (α*_0_* = *Ṁ*O_2_/*P*O_2_) throughout the trial. Stingray illustration by Sophia Emmons.

**Figure 4:**
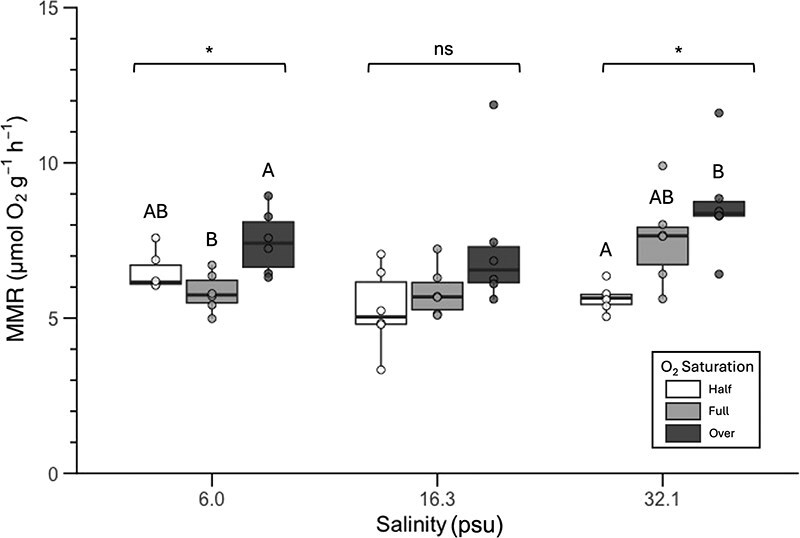
Box plot of maximum oxygen uptake rate (MMR) by salinity (psu) between saturation groups. White boxes are half air-saturation (11.7 kPa), light grey boxes are air saturation (20.4 kPa) and dark grey boxes are over air-saturation (27.0 kPa). Horizontal bar within each box is the median for each group. The bottom and top of each box represents the 25th and 75th percentiles for each group, respectively, and the bottom and top whiskers represent the minimum and maximum stingray MMR for each group, respectively. Corresponding points represent individual values from each MMR trial. Significant differences in MMR between saturation groups is indicated above brackets for each salinity (* = significant difference and ns = no significant difference). Different letters (**A** and **B**) indicate significant differences in MMR between saturation groups based on Tukey’s *post hoc* test. Letters apply only within each salinity group and do not denote differences across salinities. Summary statistics for these data can be found in Table 2 and hypothesis testing results can be found in Supplementary Tables S5.

In the hypoxia portion of the trial, *Ṁ*O_2_ was calculated as the negative slope of a linear least-squares regression of *P*O_2_ as a function of time for all data within each sequential bin and normalized for chamber volume and animal mass as described above to have units of μmol O_2_ g^−1^ hr^−1^ (Supplementary Fig. S1). As previously noted (Prinzing *et al.*, 2021), MMR and related metrics are sensitive to the averaging period due to slight differences between trials in activity, trial duration and chamber size as well as random noise. We analyzed the effect of bin size on metabolic metrics and found that, as expected (Prinzing *et al.*, 2021), oxygen supply capacity was elevated and variable at very small bin sizes but stabilized at some sufficiently large bin width specific to each trial. Beyond that sufficiently large bin size, the choice of bin size had a negligible effect on α and metabolic rates. Consequently, a bin size value that minimized both α and the standard deviation of the three α_0_ values used in its calculation, was selected from this stable region for each trial.

Oxygen supply (α_0_) was calculated for each bin as *Ṁ*O_2_/*P*O_2_, where *P*O_2_ is the average oxygen pressure recorded during the bin (Seibel *et al.*, 2021). At MMR, for a given *P*O_2_, oxygen supply is maximized and reaches capacity (α). The three highest α_0_ from the hypoxia trial were averaged to estimate α for each stingray (Fig. 3). Critical oxygen partial pressure (*P*_cMax_) was calculated for each individual using the α value from their associated hypoxia trial and the highest MMR value across all saturation trials:


(2) \begin{equation*} {P}_{c\operatorname{Max}}=\frac{\mathrm{MMR}}{\mathrm{\alpha}} \end{equation*}

**Table 2:** Atlantic stingray (*H. sabinus*) trial summaries

Salinity group	High	Medium	Low
Salinity (psu)	32.09 ± 1.35	16.34 ± 0.75	5.93 ± 0.25
Temperature (°C)	26.14 ± 0.06	25.96 ± 0.05	26.01 ± 0.04
**kPa (% air saturation)**	**MMR (μmol O**_**2**_ **g**^**−1**^** h**^**−1**^**)**		
11.71 **±** 1.59 (56%)	5.64 ± 0.44	5.29 ± 1.32	6.49 ± 0.69
20.39 **±** 0.83 (97%)	7.54 ± 1.37	5.85 ± 0.81	5.83 ± 0.61
27.02 **±** 1.88 (128%)	8.65 ± 1.67	7.35 ± 2.30	7.46 ± 1.03
**Oxygen supply capacity (μ mol O**_**2**_ **g**^**−1**^**h**^**−1**^**kPa**^**−1**^**)**			
α (from hypoxia trial)	0.56 ± 0.05	0.60 ± 0.03	0.68 ± 0.02
**Estimated** ***P***_**cMax**_ **(kPa)**			
MMR*/*α	16.17 ± 1.58	12.44 ± 1.27	11.19 ± 0.80

Measurements are reported as mean ± standard deviation. Critical upper oxygen limit (*P*_cMax_) was calculated based on MMR values at specific to each individual’s oxygen supply capacity (α) and MMR.

**Figure 5:**
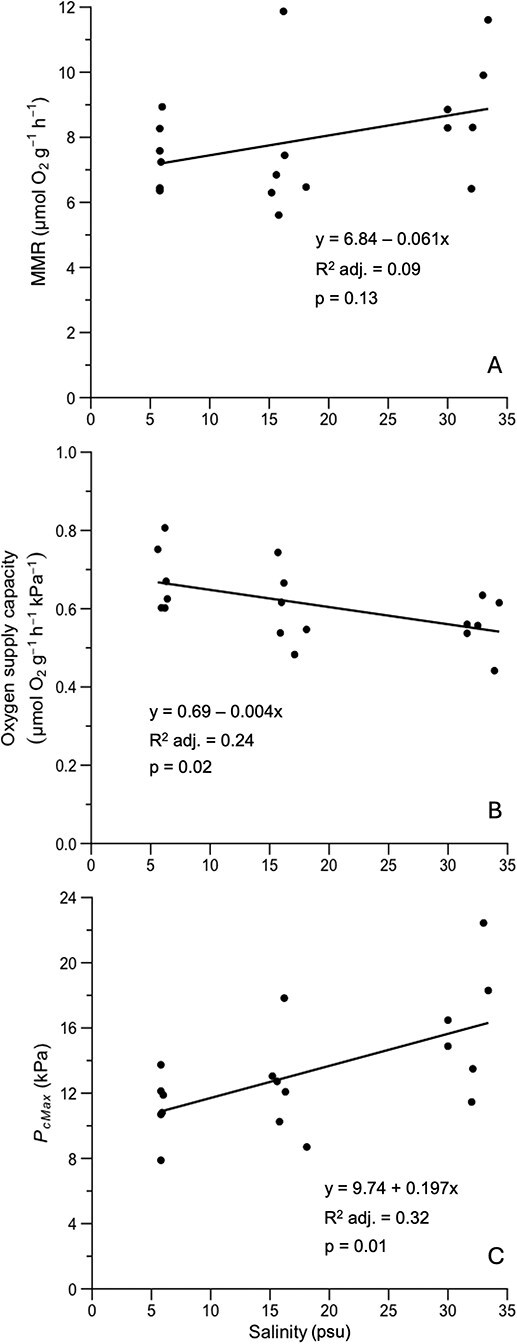
Linear regressions between salinity (psu) and MMR (μmol O^2^ g^−1^ h^−1^) (**A**), oxygen supply capacity (μmol O^2^ g^−1^ h^−1^ kPa^−1^) (**B**) and upper critical oxygen limit (*P*_cMax_; kPa) [**C**]. Note differences in *y*-axis units and values between graphs that affect the visual depiction of the trendline slope. For statistical analysis, see Supplementary Tables S6–S11.

### Statistical analyses

All analyses were performed in R and determinations of significance (*P* < 0.05) were based on 10,000 randomized permutations of the raw data to generate null-scenario *P*-values (unless otherwise noted). Due to the unequal distribution of sex within salinity levels (Table 1), these analyses excluded the effect of sex and its interacting effect with salinity. Any significant results found in this study are unlikely to be driven by sexual physiological differences given that nine individuals were sexually immature (<218 g), and the sexually mature individuals were separated by sex to prevent fertilization while in captivity. Across all levels of grouping factors based on salinity and *P*O_2_, the MMR *P*_cMax_ and oxygen supply capacity data were tested for normality using the Lilliefors test (Supplementary Table S1; Yap and Sim, 2011; R Core Team, 2024) and for homogeneity of variance using Levene’s test (Supplementary Table S2; Glass, 1966; Fox and Weisberg, 2019). Accounting for each salinity level individually, one-way analysis of variance (ANOVA) tests were performed to test for mean differences in mean MMR among *P*O_2_ treatments. In cases where ANOVA results were deemed significant, a *post hoc* Tukey test was run to determine which groups differed from one another (R Core Team, 2024). Linear regressions were performed to test for significant effects of salinity (psu) on MMR, oxygen supply capacity and *P*_cMax_ (R Core Team, 2024). The effect of salinity on these metabolic parameters is likely a proportional response and thus was analyzed to reflect the continuous nature of both the independent and dependent variables. Salinity was identified as a predictor of each dependent variable if linear regression results were significant.

## Results

Eighteen Atlantic stingrays were used across three salinity treatments (*n* = 6 per treatment; 160–1036 g wet mass, mean 564 g ± 341 SD, Supplementary Fig. S2). The sex ratio (7F:11M) was not equally distributed within or between salinity groups, specifically, the low-salinity sampling collections returned zero female stingrays (Table 1). Data for MMR, oxygen supply capacity and *P*_cMax_ were normally distributed (Supplementary Table S1), however, and met the homogeneity of variance assumptions (Supplementary Table S2) to run parametric tests.

### M‌MR

Microbial respiration was less than 5% of the total rate in all trials. In stingrays exposed to high or low salinity water, *P*O_2_ had a significant effect on mean MMR observed (Fig. 4; Table 2; Supplementary Tables S3–S5). In high salinity water, the mean MMR of stingrays at 11.7 kPa (5.64 ± 0.44 SD μmol O_2_ g^−1^ h^−1^) was significantly lower than at 27 kPa (8.65 ± 1.67 SD μmol O_2_ g^−1^ h^−1^; one-way ANOVA: *F* = 8.06, *P* = 0.004; Tukey’s *post hoc*: *Q* = 5.62, *P* = 0.003). In low salinity water, the mean MMR of stingrays at 27 kPa (7.46 ± 1.03 SD μmol O_2_ g^−1^ h^−1^) was significantly higher than at 21 kPa (5.83 ± 0.61 SD μmol O_2_ g^−1^ h^−1^; one-way ANOVA: *F* = 6.70, *P* = 0.008; Tukey’s *post hoc*: *Q* = 5.15, *P* = 0.006). There was no significant difference in MMR between saturation levels at the medium salinity (one-way ANOVA: *F* = 2.66, *P* = 0.103). Linear regression analysis revealed that salinity did not predict MMR better than chance alone, and the model’s slope was not considered significantly different from zero (*F* = 2.62, *P* = 0.13; Fig. 5A; Table 3; Supplementary Tables S6 and S7).

### Oxygen supply capacity

Salinity was found to be a significant predictor for α; as salinity decreased, α significantly increased (*F* = 6.34, *P* = 0.023; Table 3; Supplementary Tables S8 and S9). In high salinity, stingrays had a mean α of 0.56 (± 0.12 SD) μmol O_2_ g^−1^h^−1^kPa^−1^ compared to 0.68 (± 0.05 SD) μmol O_2_ g^−1^h^−1^kPa^−1^ at low salinity (Table 2). This means that Atlantic stingrays are more effective at supplying oxygen to their bodies under low salinity compared to high-salinity marine environments (Fig. 5B).

### Upper critical oxygen limit

**Figure 6:**
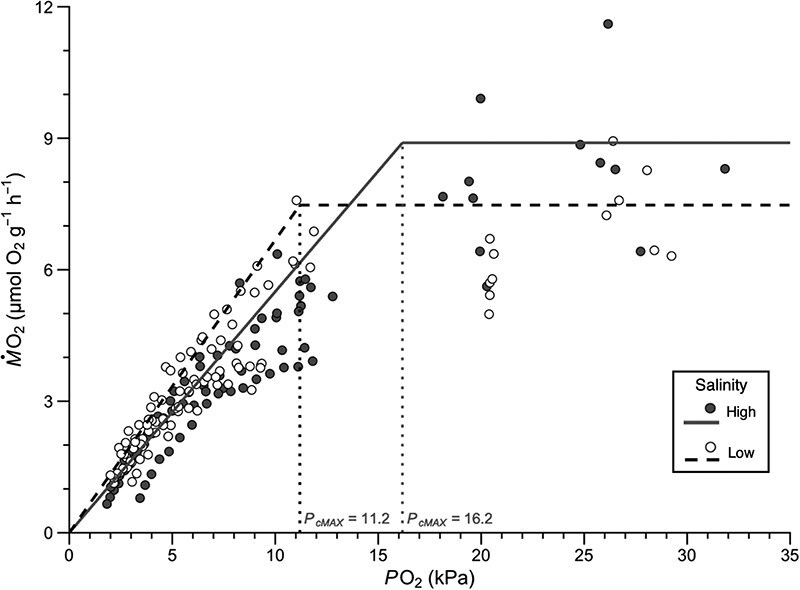
Metabolic rate (*Ṁ*O_2_) of Atlantic stingray (*H. sabinus*) high salinity (32 psu; dark grey) and low salinity (6 psu; white) as measured across a range of oxygen partial pressures (*P*O_2_, kPa). *Ṁ*O_2_ measurements from both MMR and hypoxia trials were used. Critical oxygen threshold (*P*_cMax_), below which metabolism becomes oxygen-limited, is marked by vertical dotted lines for each salinity group: 16.2 ± 1.6 kPa for high salinity and 11.2 ± 0.8 kPa for low salinity. Dashed and solid lines from 0 kPa to *P*_cMax_ values represent average oxygen supply capacity values at low and high salinity, respectively. Dashed and solid horizontal lines from *P*_cMax_ to 32 kPa reflect the average MMR at low and high salinity, respectively. Data revealed that Atlantic stingrays become oxygen-limited at a lower *P*O_2_ under low salinity conditions when compared to high salinity.

The *P*_cMax_ was calculated from the intersection of MMR and the line of limiting oxygen with the slope equal to the α. The mean *P*_cMax_ in stingrays exposed to low salinity (11.2 ± 1.96 SD kPa) was lower than at medium (12.4 ± 3.11 SD kPa) and high salinity (16.2 ± 3.87 SD kPa; Table 2). In a linear model, salinity significantly predicted *P*_cMax_ (adjusted *R*^2^ = 0.32; *F* = 8.95, *P* = 0.009; Table 3; Figs 5C and 6; Supplementary Tables S10 and S11). The *P*_cMax_ increased significantly with salinity, revealing that Atlantic stingrays are more tolerant to low oxygen when in low salinity (Fig. 5C).

## Discussion

The aim of this study was to evaluate the capacity of the Atlantic stingray to supply and utilize oxygen across a range of salinities to gain insight into its physiological plasticity. This study presents the first estimates of oxygen supply capacity and metabolic rates in this species, revealing that it physiologically enhances hypoxia tolerance at low salinities. These findings indicate that at high salinities (≈ 32 psu), the Atlantic stingray becomes oxygen-limited at approximately 77% of air saturation (i.e. *P*_cMax_ = 16.2 kPa; Table 2), situating it at the upper range of values reported for other estuarine species (8–15 kPa; Seibel, 2023). In contrast, marine species typically exhibit higher *P*_cMax_ values near air saturation (range 18–23 kPa; Seibel and Deutsch, 2020). At low salinities (≈ 6 psu), the Atlantic stingray becomes oxygen limited at 55% of air saturation (i.e. *P*_cMax_ = 11.2 kPa; Table 2), aligning more closely with other estuarine and freshwater (*P*_cMax_ = 10 kPa) species (Seibel and Deutsch, 2020; Seibel, 2023). Notably, in low salinity conditions, Atlantic stingrays increase their oxygen supply capacity and reduce *P*_cMax_ (Fig. 6), suggesting an enhanced ability to maintain metabolic activity under low oxygen conditions. This physiological plasticity may represent an adaptive response to fluctuating oxygen and salinity conditions in estuarine environments (Kuo and Neilson, 1987; Yin *et al.*, 2004) and highlights the potential role of salinity-linked oxygen dynamics in shaping the physiological responses of euryhaline species in coastal ecosystems.

Salinity changes are known to influence the oxygen cascade, potentially driving the observed variations in oxygen supply capacity in the Atlantic stingray. For example, blood-oxygen binding is sometimes sensitive to salinity fluctuations, though it is highly dependent on the species’ osmoregulatory mechanisms (Magnum, 1983). Elasmobranchs, including stingrays, retain urea and trimethylamine N-oxide (TMAO) in their blood to achieve osmotic balance with their environment (Shuttleworth, 1988). This complex balance between osmolarity, pH and osmolytes like urea and TMAO influences blood-oxygen binding in varied ways across elasmobranch species (Weber, 1983; Morrison *et al.*, 2015; Ballantyne, 2016). In some elasmobranchs, blood-oxygen binding is unaffected by changes in urea and TMAO concentrations (Burke, 1974; Mumm *et al.*, 1978); whereas, other species show a reduction in oxygen binding affinity at lower salinities (Cooper and Morris, 2004). In the Atlantic stingray, haemoglobin (Hb) oxygen binding is largely unaffected by urea or any other osmolyte concentrations (Mumm *et al.*, 1978). A similar pattern is seen in another euryhaline elasmobranch, the bull shark (*Carcharhinus leucas*), which also maintains Hb-O_2_ affinity across salinity gradients (Burke, 1974). However, the marine Port Jackson shark (*Heterodontus portusjacksoni*) shows a decrease in Hb-O_2_ affinity as salinity increases (Cooper and Morris, 2004). The effects of TMAO on blood-oxygen transport are less studied, but in crustaceans, TMAO has been shown to decrease the oxygen affinity of the oxygen transport protein haemocyanin (Sanders *et al.*, 1992). The variability in physiological responses to salinity changes may be driven by differences in species’ life histories, activity levels, or the primary environments they inhabit. The salinity independence of the Atlantic stingray and bull shark oxygen-binding is likely advantageous in their dynamic native environments. However, the mechanism promoting increased oxygen supply under low salinity conditions remains unexplained.

**Table 3:** Summary of linear regression results for dependent variables plotted against salinity (psu)

Dependent variable	*b*_1_	*t*	*P_t_*	*R* ^2^	*R* ^2^ adj	*F*	*P_F_*
α	−0.004	−2.518	0.023^*^	0.2838	0.2391	6.3414	0.023^*^
*P*_cMax_	0.197	2.975	0.009^*^	0.3561	0.3158	8.8491	0.009^*^
MMR	0.061	1.618	0.125	0.1405	0.0868	2.6162	0.125

For each regression, *b*_1_ = regression slope, *t* = *t*-test statistic, *F* = *F*-test statistic and their associated *P*-values (*P_t_* and *P_F_*, respectively). A significant *P*-value (α = 0.05) is indicated by a ^*^. The *R*^2^ value is the fraction of the total variation in the dependent variable that is explained by salinity, and the adjusted *R*^2^ value (*R*^2^ adj). Dependent variables are α = oxygen supply capacity (μmol O_2_ g^−1^ h^−1^ kPa^−1^), *P*_cMax_ = upper critical oxygen limit (kPa) and MMR = maximum metabolic rate (μmol O_2_ g^−1^ h^−1^).

Salinity was not a significant predictor of MMR (Fig. 5A), indicating that MMR remained stable across all tested salinities. This suggests that any energetic costs or savings associated with changes in urea and TMAO synthesis and retention due to salinity fluctuations are not detectable during periods of high physical activity. Urea and TMAO synthesis and retention are estimated to account for 10–15% of SMR in elasmobranch species (Kirschner, 1993), suggesting euryhaline species may experience lower metabolic costs while at rest in low salinity environments because urea retention is reduced (Deck *et al.*, 2016). In the Atlantic stingray, for example, when freshwater populations are acclimated to high salinity, plasma urea and other osmolyte concentrations significantly increase, indicating an upregulation of urea retention as salinity rises and potentially signalling an increase in metabolic costs (Piermarini and Evans, 1998). However, during periods of peak physical activity, *Ṁ*O_2_ is above SMR because of increased locomotion, or in this case, excess post-exercise oxygen consumption. There is evidence in euryhaline teleosts that osmoregulation can impact MMR, increasing MMR outside of isosmotic environments and accounting for 20–30% of total metabolism (Rao, 1968). Results from this study suggest that if there are metabolic costs incurred by urea and TMAO synthesis and retention, they are not detectable at MMR.

Different salinity environments present varying ecological pressures that may influence metabolic capacity in ways not measured in this study. In many estuarine systems, the Atlantic stingray encounters relatively few predators; key threats such as alligators (*Alligator mississippiensis*) and bull sharks are generally limited (Nifong and Lowers, 2017). In contrast, marine habitats present a greater variety of predators, including other elasmobranchs such as blacktip (*C. limbatus*) and hammerhead (*Sphyrna* spp.) sharks (Hoffmayer and Parsons, 2003). These differences in predation pressure could influence metabolic demands across salinity gradients, though this study did not detect differences in MMR. Salinity may be the proximal trigger for adjustments to oxygen supply and demand. For example, in oceanic vertical migrators, deep daytime habitats and shallow nighttime habitats offer stark differences in predation pressure as well as oxygen and temperature. Temperature was proposed as the trigger for physiological changes required to meet the divergent challenges of that day and night habitats (Seibel and Birk, 2022). Salinity may act as a similar queue for some euryhaline species, depending on their interspecies interactions.

The findings of this study reflect a 24-h exposure to salinity treatments representative of short-term fluctuations in salinity and dissolved oxygen within estuarine environments (Snelson *et al.*, 1988; Schmidt *et al.*, 2004). Short-term metabolic responses to salinity changes have been observed in other elasmobranchs, such as gummy (*Mustelus antarcticus*) and school (*Galeorhinus galeus*) sharks, where hypersalinity exposure (>35 psu) resulted in an initial increase in routine metabolic rate (>35 psu; Tunnah *et al.*, 2016). In contrast, no change in MMR was observed in Atlantic stingrays under hyposaline conditions in this study. This discrepancy suggests that the energetic demands of osmoregulation may differ depending on the direction of salinity change (hypo- vs hypersalinity) and that species-specific physiological adaptations may influence metabolic responses. These divergent patterns highlight the complexity of metabolic adjustments associated with salinity-driven shifts in urea, TMAO concentrations, as well as Hb-O_2_ affinity. Studies on other elasmobranch species indicate that metabolic rates often return to baseline levels following prolonged exposure to altered salinities, typically within 72 h to two weeks (Cooper and Morris, 2004; Tunnah *et al.*, 2016). Future research should assess whether Atlantic stingrays exhibit a similar trend, as this would suggest that reductions in metabolic capacity under low salinity conditions are temporary rather than sustained.

The ability of Atlantic stingrays to enhance their oxygen supply capacity under low-salinity conditions suggests a degree of resilience to environmental fluctuations. However, whether they can tolerate the increasing environmental pressures resulting from anthropogenic climate change remains uncertain. Even if Atlantic stingrays successfully adapt or acclimate to these changes, shifts in prey distribution and availability could alter their ecological roles if other estuarine species lack similar tolerances. As key contributors to nutrient cycling and sediment oxygenation via bioturbation (Grew *et al.*, 2025), stingrays perform essential ecosystem functions that may be compromised if these changing abiotic render their habitats unsuitable or disrupt prey availability.

This study highlights the critical role of salinity in modulating oxygen supply capacity and thus hypoxia tolerance, an adaptation that is commonly enhanced in estuarine species relative to their marine counterparts (Seibel, 2023). Future research should investigate potential interactions between salinity and temperature, as temperature strongly influences both oxygen supply and demand (Alfonso *et al.*, 2021). Given that estuarine environments frequently experience simultaneous fluctuations in multiple abiotic conditions, a more integrative approach is needed to fully understand the physiological responses of organisms to these complex environmental stressors. Physiological metrics are increasingly being used to predict species’ shifting biogeographies, oxygen limitation of body size and potential fisheries impacts of ocean warming and deoxygenation (Cheung *et al.*, 2013; Deutsch *et al.*, 2015; Rubalcaba *et al.*, 2020; Deutsch *et al.*, 2022; Parouffe *et al.*, 2023; Slesinger *et al.*, 2024). These physiological models have been challenged recently (Seibel, 2024), in part because they do not incorporate environmental acclimation or plasticity of metabolic traits, such as that observed here. Advancing knowledge of these adaptive mechanisms will improve predictions of estuarine species’ resilience in the face of ongoing climate and ecological change.

### Ethical declarations

Collection of *H. sabinus* was conducted under Special Activities Licence granted by the state of Florida (SAL-22-2143-SR). All experiments were conducted under IACUC protocol #W IS00011888.

## Acknowledgements

We are grateful to the University of South Florida for providing the facilities that allowed for the maintenance of the animals and execution of experiments, with special thanks to Garrett Miller and Michael Schram. Thanks go to Tracy Shaw, Alyssa Andres, Katie Lynch and Sarah Schaal for their help with specimen collection, husbandry and respirometry trials.

## Author contributions

S.M.E. (Conceptualization, Investigation, Data curation, Formal Analysis, Visualization, Writing—original draft), J.L.R. (Supervision, Writing—review & editing), J.P.K. (Methodology, Formal Analysis, Writing—review & editing), M.A.P. (Data curation; Writing—review & editing), A.W.T. (Methodology, Writing—review & editing), C.A.S. (Supervision, Writing—review & editing), B.A.S. (Supervision, Resources, Project administration, Formal Analysis, Visualization, Writing—review & editing).

## Conflicts of interest

J.L.R. is an Associate Editor for Conservation Physiology but had no involvement in the peer review process for this paper.

## Funding

This work was funded by the Graduate Student Success Fellowship with the University of South Florida.

## Data availability

Data available upon request.

## Supplementary material

Supplementary material is available at *Conservation Physiology* online.

## Supplementary Material

Web_Material_coaf071
