# Supplementary material for: Euryhaline Atlantic stingray (Hypanus sabinus) exhibit elevated oxygen supply capacity in hyposaline water: implications for estuarine species resilience and conservation
Source: Conserv Physiol. 2025 Oct 21;13(1):coaf071. doi: 10.1093/conphys/coaf071 (PMC12539869; doi:10.1093/conphys/coaf071)
Supplement: Web_Material_coaf071 [file web_material_coaf071.zip › Emmons_Euryhaline_SuppMat.pdf]

Supplementary Material

Euryhaline Atlantic stingray (*Hypanus sabinus*) exhibit elevated oxygen supply capacity in hyposaline water

Sophia M. Emmons[1,2], Jodie L. Rummer[1], Joshua P. Kilborn[2], Maria Pierce[3], Alexander W. Timpe[2], Colin A. Simpfendorfer[1], Brad A. Seibel[2]

[1]College of Science and Engineering, James Cook University, Townsville, Queensland, 4811 Australia.
[2]College of Marine Science, University of South Florida, St. Petersburg, Florida 33701 United States.
[3]Marine Science Department, Eckerd College, St. Petersburg, Florida 33711 United States.

Corresponding author: Sophia Emmons, sophia.emmons@my.jcu.edu.au, ORCID: 0000-0003-3918-4647

Supplementary Table 1 Reported statistics for Lilliefors test for normality. MMR is maximum metabolic rate, PO_2 is partial pressure of oxygen, α is oxygen supply capacity, and P_{cMax} is partial pressure of oxygen at which point MMR becomes oxygen dependent. High salinity is 32 ppt, medium salinity is 16 ppt, and low salinity is 6 ppt. *L* is the test statistic, and *p* is the significance value (alpha ≤ 0.05)

Lilliefors	MMR by PO_2 (kPa)			MMR by salinity groups			α	P_{cMax}
	11.7	*20.4*	*27.0*	*High*	*Medium*	*Low*		
L	0.10	0.19	0.15	0.19	0.18	0.17	0.12	0.17
p	0.91	0.09	0.33	0.10	0.14	0.18	0.66	0.18

Supplementary Table 2 Reported statistics for Levene's test for Homogeneity of Variance. MMR is maximum metabolic rate, PO_2 is partial pressure of oxygen, α is oxygen supply capacity, and P_{cMax} is partial pressure of oxygen at which point MMR becomes oxygen dependent. High salinity is 32 ppt, medium salinity is 16 ppt, and low salinity is 6 ppt. *F* is the test statistic, df_{Group} is the degrees of freedom per number of groups, df_{Error} is the degrees of freedom from within group variability, and *p* is the significance value (alpha ≤ 0.05). "MMR within PO_2 (kPa)" tests for homogeneity of variance among salinity groups at each PO_2, and "MMR within salinity groups" tests among PO_2 levels within each salinity group

Levene's test	MMR within PO_2 (kPa)			MMR within salinity groups			α	P_{cMax}
	11.7	*20.4*	*27.0*	*High*	*Medium*	*Low*		
F	2.09	0.97	0.26	0.99	0.61	1.11	0.58	0.91
df_{Group}	2	2	2	2	2	2	2	2
df_{Error}	15	15	15	15	15	15	15	15
p	0.16	0.40	0.78	0.39	0.56	0.35	0.57	0.42

Supplementary Table 3 One-way ANOVA showing changes in maximum metabolic rate (MMR) at high salinity (32 ppt) depending on starting oxygen saturation point. For each source of variability, *df* = degrees of freedom, *SS* = sum of squares, and *MS* = mean squares. The *F*-statistic is used to assess differences in the mean MMR among the *h* = one-half (11.7 kPa), *f* = full (20.4 kPa), *o* = over (27.0 kPa) air saturation, and * indicates a significant *p*-value (alpha = 0.05). Results of *post*-hoc Tukey tests used for pairwise comparisons among salinity are also presented

**Source**	***df***	***SS***	***MS***	***F***	***p***
O_2 Saturation	2	27.743	13.872	8.063	0.004*
Residual	15	25.805	1.720		
Total	17	53.548			
Comparison	**Tukey's *Q***	***p***			
Saturation Pairs					
h-f	3.545	0.059			
h-o	5.615	0.003*			
f-o	2.070	0.335			

Supplementary Table 4 One-way ANOVA testing for differences in the mean MMR at medium salinity (16 ppt) among oxygen saturation points (kPa). See Supplementary Table 3 for details and definitions for ANOVA test

**Source**	***df***	***SS***	***MS***	***F***	***p***
O_2 Saturation	2	13.676	6.838	2.66	0.103
Residual	15	38.53	2.57		
Total	17	52.207			

Supplementary Table 5 One-way ANOVA testing for differences in the mean MMR at low salinity (6 ppt) among oxygen saturation points (kPa). See Supplementary Table 3 for details and definitions for ANOVA and *post hoc* Tukey tests

**Source**	***df***	***SS***	***MS***	***F***	***p***
O_2 Saturation	2	8.137	4.068	6.702	0.008*
Residual	15	9.106	0.607		
Total	17	17.243			
Comparison	**Tukey's *Q***	***p***			
Saturation Pairs					
h-f	2.071	0.335			
h-o	3.074	0.109			
f-o	5.145	0.006*			

Supplementary Table 6 Linear regression analysis of salinity and MMR. For each source of regression, *SS* = sum of squares, *df* = degrees of freedom, and *MS* = mean squares. The *F*-statistic is the ratio of the variance explained to unexplained

	SS	***df***	***MS***	***F***	***p***
Regression	7.638	1	7.638	2.616	0.125
Residual	46.711	16	2.919		
Total	54.349	17			

Supplementary Table 7 Summary of coefficients for linear regression analysis of maximum metabolic rate (MMR*;* µmol O_2 g^{-1} h^{-1}) plotted against salinity (ppt)

	Coefficients	*SE*	*t*	p_t	Lower 95%	Upper 95%
Intercept	6.837	0.787	8.690	<0.001	5.169	8.505
Salinity	0.061	0.037	1.617	0.125	-0.019	0.141

Supplementary Table 8 Linear regression analysis of salinity and oxygen supply capacity (α; µmol O_2 g^{-1} h^{-1} kPa^{-1}). A significant *p*-value (alpha = 0.05) is indicated by a *. See Supplementary Table 7 for details and definitions for linear regression analyses

	SS	*df*	*MS*	*F*	*p*
Regression	0.042	1	0.042	6.341	0.023*
Residual	0.106	16	0.007		
Total	0.149	17			

Supplementary Table 9 Summary of coefficients for linear regression analysis of oxygen supply capacity (α; µmol O_2 g^{-1} h^{-1} kPa^{-1}) plotted against salinity (ppt)

Predictor	Coefficients	*SE*	*t*	p_t	Lower 95%	Upper 95%
Intercept	0.692	0.038	18.445	<0.001	0.612	0.772
Salinity	-0.004	0.002	-2.518	0.023	-0.008	-0.001

Supplementary Table 10 Linear regression analysis of salinity and upper critical oxygen limit (P_{cMax}; kPa). A significant *p*-value (alpha = 0.05) is indicated by a *. See Supplementary Table 6 for details and definitions for linear regression analyses

	SS	*df*	*MS*	*F*	*p*
Regression	79.479	1	79.479	8.849	0.009*
Residual	143.710	16	8.982		
Total	223.189	17			

Supplementary Table 11 Summary of coefficients for linear regression analysis of upper critical oxygen limit (P_{cMax}; kPa) plotted against salinity (ppt)

	Coefficients	*SE*	*t*	p_t	Lower 95%	Upper 95%
Intercept	9.742	1.380	7.059	<0.001	6.816	12.667
Salinity	0.197	0.066	2.975	0.009	0.337	0.057

Supplementary Table 12 Criteria for reporting intermittent-flow respirometry results to estimate maximum metabolic rate (MMR) and critical oxygen limits at hypoxia based on checklist developed by Killen et al., 2021.

**Item**	**Description**
Animal body mass	See Table 1
Chamber volume	See Table 1
Chamber mixing	Achieved using off-set recirculatory pump (Fig. 2)
Chamber to body size ratio (L:kg)	Largest: 133.0<br>Smallest: 38.6
Tubing volume used in MO_2 calculations	Yes
Respirometer material	Acrylic
Oxygen data recording	Firesting Optical Oxygen and Temperature Meter, PyroScience Robust Oxygen Probe OXROB10 (PyroScience, gmbh, Aachen, Germany)
Sampling frequency	Every 3 seconds
Probe placement	In recirculatory circuit
Chamber flushing	Duration to trial PO2: 3-5 mins (dependent on chamber size)
Measure/flushing cycles	Measure: 15 mins<br>Flush: 3-5 mins (to trial starting PO2)
Delay time excluded from measurement cycles	1.5 minutes
Probe calibration	Probes were recalibrated before every trial. Calibrated to 0% using OXCAL 0% O2 Calibration Capsules (PyroScience, gmbh, Aachen, Germany) dissolved in deionized water. Calibrated to 100% using experimental water bubbled to normoxia.
Temperature compensation	No temperature compensation was used during the recording of oxygen concentration. Temperature was recorded using a Temperature probe and Firesting Optical Oxygen and Temperature Meter, (PyroScience, gmbh, Aachen, Germany).
Experimental temperature	26ºC (Table 2)
Temperature controls	Aquarium heaters (Methods section 2.2)
Photoperiod	Day
Temperature bath maintenance	Drained and cleaned between each trial using a 5% bleach solution then rinsed with deionized water.
Temperature bath volume	~500L
Minimum PO2 reached during trials	1.7 kPa (8% air saturation)
External disturbance	Chambers were not shielded from visual disturbance because SMR/RMR was not measured in this study.
# animals per temperature bath	One animal was measured at a time, in one temperature bath.
Animal fasting	Animals were fasted for 48 hours prior to respirometry trials (Di Santo and Bennett, 2011)
Acclimation time to lab conditions	Animals were acclimated to lab conditions for 1-1.5 weeks before undergoing respirometry trials. Animals were considered acclimated when they consistently ate when offered food.
Microbial respiration	Microbial respiration was accounted for by measuring the oxygen depletion in the closed respirometry chamber for one hour following each trial. Slopes were modelled linearly and removed from calculations using the R package "respirometry" (Birk, 2024).
% microbial respiration	<8% across all trials

Chamber cleaning	Respirometry chambers (and tubing etc) were cleaned between every trial using a 5% bleach solution and rinsed with deionized water.
MMR exhaustive protocol	Animals were chased at normoxia until they no longer responded to physical stimuli with burst-swimming. Animals were then repeatedly ventrally inverted and until they could no longer right themselves. Animals were then given one minute of air exposure before being placed inside the respirometry chamber (Fig. 2)
MMR calculation	Within the measurement period with the fastest declining oxygen %, MMR was averaged across the three bins (45 seconds, consecutive) with the highest MO2 values using the "respirometry" R package (Birk, 2024).
Sample size	See Table 1
Data calculation	R package "respirometry" (Birk, 2024). See Methods section 2.5
Body-mass accounting	Body mass of the animal was accounted for in the net respirometry chamber volume calculations and in the data analysis phase by using a body-mass scaling coefficient calculated in the R package "respirometry" (Birk, 2024). See Methods section 2.5.

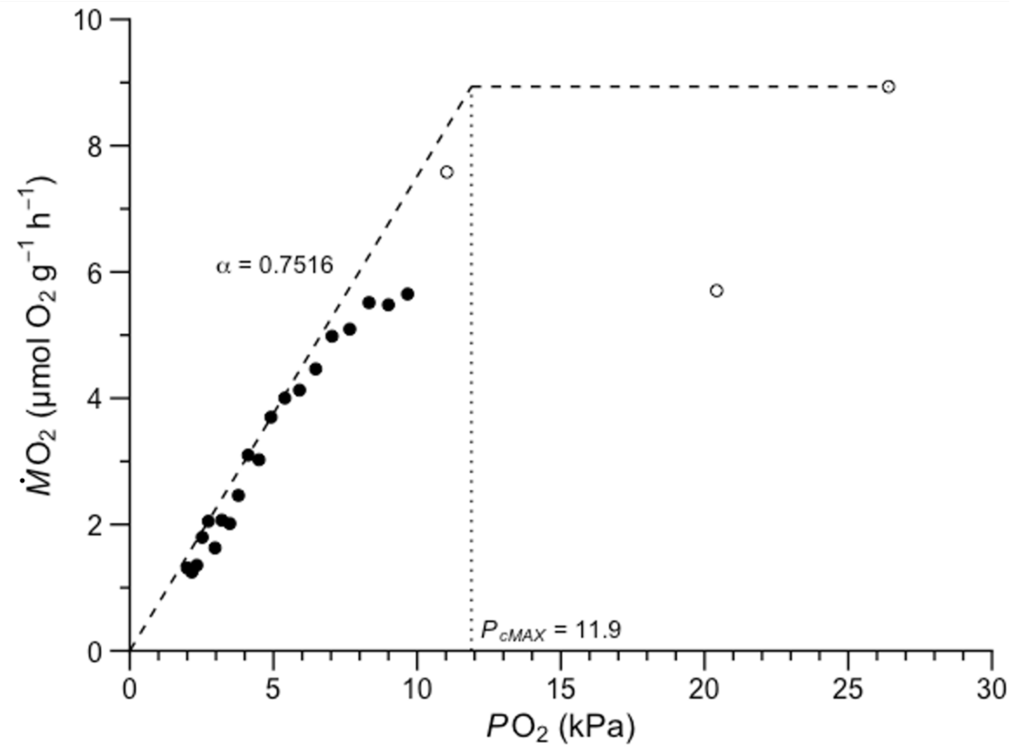

Supplementary Fig. 1 Representative oxygen uptake rate ($\dot{M}O_2$) data for a single stingray in low salinity. Open circles indicate maximum $\dot{M}O_2$ values from maximum metabolic rate (MMR) trials conducted at three oxygen partial pressures (PO_2). Black circles represent $\dot{M}O_2$ values from the hypoxia trial. The sloped dashed line depicts the oxygen supply capacity (α) for this individual, which plateaus into a horizontal dashed line, indicating the MMR. The PO_2 at which this individual's metabolic rate becomes oxygen-dependent (P_{cMax}) is 11.9 kPa

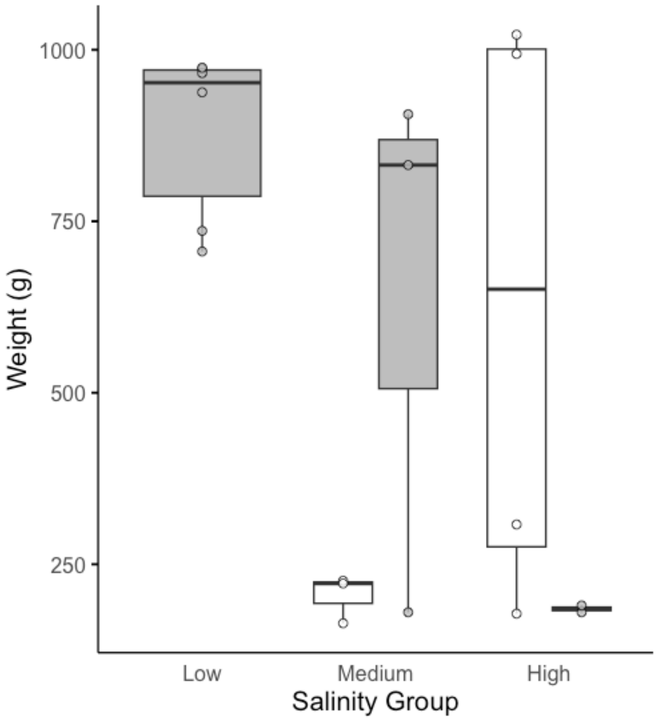

Supplementary Fig. 2 Distribution of stingray wet weight (g) by sex across salinity groups. White boxes and points are female; grey boxes and points are male. Salinity groups represent low (6 ppt), medium (16 ppt) and high (32 ppt) salinity treatments. There were no females present in the low salinity group. Means and standard deviations can be found in Table 1
